# Correlates of screen time among 8–19-year-old students in China

**DOI:** 10.1186/s12889-018-5355-3

**Published:** 2018-04-10

**Authors:** Sunyue Ye, Lijian Chen, Qineng Wang, Qinggong Li

**Affiliations:** 1 Chronic Disease Research Institute, School of Public Health, School of Medicine, Zhejiang University, 866 Yu-hang-tang Road, Hangzhou, 310058 China; 2 Physical Activity and Health Research Institute, Zhejiang Financial College, Hangzhou, 310018 China; 3 Dongcheng Middle School, Hangzhou, 310019 China; 4 Xiaofeng Middle School, Huzhou, 313301 China; 5 College of Teacher Education, Zhejiang Normal University, Jinhua, 321004 China

**Keywords:** Measurement, Determinants, Home environment, Parental effects, Screen-based sedentary behavior

## Abstract

**Background:**

Previous studies have shown that prolonged time spent on screen-based sedentary behavior was significantly associated with lower health status in children, independent of physical activity levels. The study aimed to explore the individual and environmental correlates of screen time (ST) among 8–19-year-old students in China.

**Methods:**

The study surveyed ST using a self-administered questionnaire in Chinese students aged 8–19 years; 1063 participants were included in the final analysis. Individual and environmental correlates of ST were assessed using a mixed-effects model (for continuous outcome variables) and multiple logistic regression model (for binary outcome variables).

**Results:**

Prolonged ST was observed in 14.7% of boys and 8.9% of girls. Of the ST, weekend and mobile phone/tablet use represented 80% and 40%, respectively. A positive relationship was observed between media accessibility and ST in both boys and girls (*p* < 0.05), whereas the presence of parents/others while using screens was a negative factor for longer ST (*p* < 0.05). Among the assessed correlates, access to a television (TV) in students’ bedrooms was associated with prolonged total and weekend ST (*p* < 0.05 and *p* < 0.001, respectively). However, spending time on a mobile phone/tablet or a computer rather than viewing a TV, along with increased media accessibility, increased ST.

**Conclusions:**

These results indicate that greater media accessibility was positively associated and the presence of parents/others was negatively associated with prolonged ST in both Chinese boys and girls. Development of new and effective strategies against prolonged ST are required, especially for small screen device-based ST on weekends.

## Background

Screen-based sedentary behavior (SSB), including many new forms of digital media, is ubiquitous and is an important part of daily activity in the current information era. A longer time spent in SSB was significantly associated with lower fitness, overweight or obesity, depression, inattention, shortened sleep duration, and cardiovascular disease risk factors in children or adolescents [1–6], independent of meeting or not meeting the recommended level of physical activity [7, 8]. Moreover, the habits of SSB developed in childhood more easily persist into adulthood compared with the habits of moderate or vigorous physical exercise [9]. The “Health Behavior in School-aged Children” project (World Health Organization) indicated that students aged 11–15 years spent more than 2 h per day viewing television (an indicator of SSB) in 30 countries, and screen time (ST) has been growing along with further popularization of new screen devices (e.g., personal computer, tablet, smart phone, etc.) [10]. A study conducted in a city in Eastern China also showed that 51.5% of adolescents spend more than 2 h on ST per day [11].

Limited previous studies have indicated that increased ST was related to male sex, being older, weekends, low parental education, and high media accessibility (such as having a television [TV] or a computer in the bedroom) [12–15]. Other studies on environmental correlates of SSB, however, have not been very clear, and some of the results were inconsistent (such as the presence of a television set in the bedroom) [16, 17]. Meanwhile, environmental correlates, such as media accessibility and parental social factors (rules or viewing accompanied by parents), play a key role in evidence-based interventions on restricting prolonged ST in school-aged children [16]. To our knowledge, few studies have explored and confirmed the modifiable correlates of prolonged ST, especially for new screen devices, such as cell phones and pads/tablets, in children in developing countries [18].

The aim of the present study was therefore to examine the correlates, including individual and environmental factors, of ST based on a validated self-reported SSB questionnaire in 8–19-year old Chinese girls and boys.

## Methods

### Participants

A total of 1164 students aged 8–19 years from five elementary, junior high, or senior high schools participated in the project from September 2015 to May 2016 in Zhejiang Province, China. We analyzed 1063 students after excluding subjects with missing variables for age or sex (*n* = 26), no informed consent (*n* = 26), or missing variables of other correlates (*n* = 49). All students and their parents provided signed informed consent forms, and the study protocols were approved by the institutional review board of Zhejiang Financial College.

### Questionnaire of SSB

The self-administered SSB questionnaire was designed based on the Adolescent Sedentary Activity Questionnaire [19] and included four items: TV or video viewing, playing for recreation or leisure on a computer, computer use for homework, and playing for recreation or leisure on a cell phone or tablet. ST, representing SSB, was defined using the same definition for sedentary screen time [20], not including physically active behavior. One question, “Do you participate in this activity?”, was changed from an answer of “daily” to include two columns: “Monday to Friday” and “Saturday to Sunday”. The intra-class correlation coefficient of the revised SSB questionnaire (Chinese version) was > 0.8 (boys: 0.81, girls: 0.85), which is considered excellent according to the recommendations of Landis et al. [21], based on an analysis of test-retest reliability.

### Correlates measurement

Environmental factors were divided into three questions of media accessibility (“Do you have a personal computer?”, “Do you have a TV in your bedroom?”, and “Do you have a cell phone/tablet?”) and two questions of parent/others social factors (“Do you have a parent or others present when you play on the computer?” and “Do you have a parent or others present when you are watching TV?”). The options of media accessibility questions were binary including “Yes” (encoded as 1) and “No” (encoded as 0). A four-point Likert scale, including “Strongly disagree” (encoded as 1), “Disagree” (encoded as 2), “Agree” (encoded as 3), and “Strongly agree” (encoded as 4), was used to measure the effects of parents or other persons. Individual information, such as sex, age, grade, and parental education level (in part), was also assessed.

### Statistical analysis

Correlates and ST are described and sex differences of variables were evaluated using the chi-squared test for categorical variables or T-test for continuous variables. Three questions of media accessibility were added as categorical variables (no screen, one screen, two screens, and three screens were 0, 1, 2, and 3, respectively) or binary variables (the values of low and high were 0–1 and 2–3, respectively) and two questions of the presence of parents/others were also added as categorical variables (strongly disagree, disagree, agree, and strongly agree were 2–3, 4, 5–6, and 7–8, respectively) or binary variables (low and high were 2–4 and 5–8, respectively). Prolonged ST was defined as ≥2 h per day, and prolonged weekend ST was defined as ≥5 h/day [22–24]. Leisure ST was defined as ST excluding time spent using the computer for school-related studying. A mixed effects model was applied to explore correlates of and interactions with ST, which included fixed effects (age, grade, media accessibility, and presence of parents/others) and random effects (schools and classes). Because of the high requirement for normality in mixed effects models, zero-mean normalization was used to transform continuous variables of age and ST previously for their non-normality. Odds ratios (OR) and 95% confidence intervals (CI) were used to describe the effects of environmental factors on ST (binary variables) based on the logistic regression model. All data were evaluated using Epidata 3.0 (double entry) and IBM SPSS 20.0 (statistical analysis); the significance level was *p* < 0.05.

## Results

### Individual characteristics in boys and girls

**Fig. 1:**
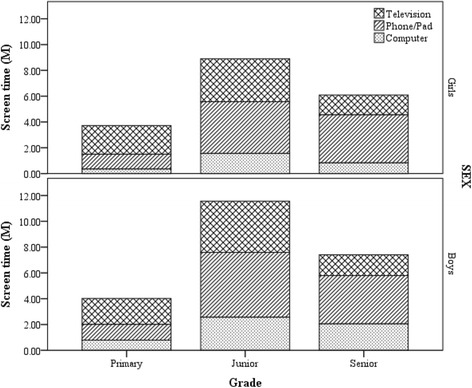
Relationship between grade and components of screen time in Chinese students

ST and leisure-based computer use were higher in boys than in girls (*p* < 0.05), and 14.7% of boys and 8.9% of girls reported prolonged ST (≥2 h/day), respectively (Table 1). Weekend ST and time spent on a phone/tablet represented about 80% and 40% of ST, respectively. The ST of junior high school students was the highest, followed by senior high school and elementary school students (*p* < 0.05) (Fig. 1). All three types of ST in junior high school students were the highest, and time spent on a phone/tablet and computer in senior high school students was higher than that of elementary school students (*p* < 0.05). TV viewing, however, was lower in senior high school students than in elementary school students (*p* < 0.05 in girls and *p* > 0.05 in boys).

**Table 1:** Characteristics of screen time in Chinese boys and girls

Variables	Boys (*n = 510*)	Girls (*n = 553*)	*P* value^a^
*Continuous variable, mean (SD)*			
Age, years	14.79(2.52)	15.05(2.49)	> 0.05
Screen time, hours/week			
Weekdays	1.86(4.85)	1.33(2.49)	< 0.05
Weekends	7.12(8.23)	5.86(5.32)	< 0.005
TV viewing			
Weekdays	0.52(1.28)	0.39(0.90)	> 0.05
Weekends	2.11(3.02)	1.91(2.25)	> 0.05
Phone/tablet use			
Weekdays	0.78(2.34)	0.62(1.67)	> 0.05
Weekends	3.01(4.54)	2.78(3.61)	> 0.05
Computer use			
Leisure			
Weekdays	0.28(1.32)	0.14(0.67)	< 0.05
Weekends	1.73(3.11)	0.87(2.17)	< 0.001
Study			
Weekdays	0.29(2.76)	0.18(0.73)	> 0.05
Weekends	0.27(0.86)	0.30(0.64)	> 0.05
*Categorical variables, /n (%)*			
Screen time ≥ 2 h/day	75(14.7)	49(8.9)	< 0.005
Grade			
Elementary school	96(18.8)	93(16.8)	> 0.05
Junior school	204(40.0)	201(36.3)	
Senior school	210(41.2)	259(46.8)	
Media accessibility			
None screen	33(6.5)	28(5.1)	> 0.05
One screens	125(24.5)	147(26.6)	
Two screens	239(46.9)	285(51.5)	
Three screens	113(22.2)	93(16.8)	
Presence of parents/others (P)			
Strongly disagree	132(25.9)	130(23.5)	> 0.05
Disagree	117(22.9)	122(22.1)	
Agree	190(37.3)	210(38.0)	
Strongly agree	71(13.9)	91(16.5)	
TV in bedroom	83(16.3)	87(15.7)	> 0.05
Computer	132(25.9)	148(26.8)	> 0.05
Phone/tablet	373(73.1)	428(77.4)	> 0.05
Presence of P while watching TV			
Strongly disagree	111(21.8)	86(15.6)	< 0.05
Disagree	123(24.1)	125(22.6)	
Agree	156(30.6)	192(34.7)	
Strongly agree	120(23.5)	150(27.1)	
Presence of P while PC use			
Strongly disagree	178(34.9)	201(36.3)	> 0.05
Disagree	179(35.1)	183(33.1)	
Agree	87(17.1)	109(19.7)	
Strongly agree	66(12.9)	60(10.8)	

^a^T-test for continuous variables and Chi-squared test for categorical variables between boys and girls, respectively.

*SD* standard deviation; *TV*, television; *P*, parents/others; *PC*, personal computer

### Environmental correlates of leisure ST

**Fig. 2:**
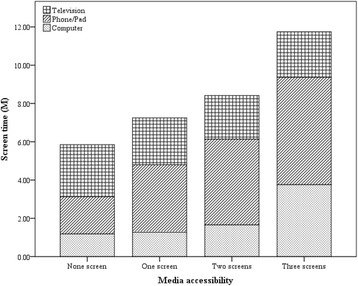
Relationship between media accessibility and components of screen time in Chinese students

ST was positively associated with media accessibility and negatively associated with presence of parents/others (*p* < 0.05) (Table 2). Time spent viewing a phone/tablet or using a computer (*p* < 0.001), but not viewing a TV (*p* > 0.05), increased along with increasing media numbers (Fig. 2). An interaction between media accessibility and the presence of parents/others was observed, and media accessibility played a more important role on prolonged ST than did the presence of parents/others (data not shown).

**Table 2:** Mixed effects models on environmental factors of screen time in Chinese boys and girls

Factors\Screen	Girls			Boys		
	ST model, Beta (95%CI)	Weekdays ST model, Beta (95%CI)	Weekends ST model, Beta (95%CI)	ST model, Beta (95%CI)	Weekdays ST model, Beta (95%CI)	Weekends ST model, Beta (95%CI)
*Media accessibility*						
None screen	Ref.	Ref.	Ref.	Ref.	Ref.	Ref.
One screens	0.16(−0.06,0.39)	0.11(− 0.13,0.35)	0.16(− 0.07,0.38)	0.30^†^(0.08,0.51)	0.14(− 0.08,0.36)	0.31^†^(0.09,0.53)
Two screens	0.42^†^(0.17,0.68)	0.24(−0.03,0.50)	0.41^†^(0.16,0.67)	0.34^†^(0.09,0.60)	0.30^*^(0.04,0.56)	0.30^*^(0.04,0.55)
Three screens	0.66^†^(0.25,1.07)	0.67^†^(0.25,1.10)	0.52^*^(0.11,0.93)	0.79^‡^(0.42,1.16)	0.68^‡^(0.30,1.07)	0.69^‡^(0.32,1.07)
*p* value for trend	< 0.005	< 0.05	< 0.05	< 0.001	< 0.001	< 0.005
*Presence of parents/others*						
Strongly disagree	Ref.	Ref.	Ref.	Ref.	Ref.	Ref.
Disagree	−0.21(−0.45,0.02)	−0.29^*^(− 0.53,-0.04)	−0.13(− 0.37,0.10)	−0.05(− 0.29,0.18)	0.02(− 0.22,0.26)	−0.08(− 0.31,0.16)
Agree	− 0.22^*^(− 0.43,-0.01)	−0.12(− 0.34,0.10)	− 0.21^*^(− 0.42,-0.01)	− 0.15(− 0.37,0.06)	− 0.11(− 0.33,0.11)	−0.15(− 0.36,0.07)
Strongly agree	− 0.35^†^(− 0.60,-0.09)	− 0.34^*^(− 0.61,-0.07)	−0.27^*^ (− 0.53,-0.01)	−0.32^*^(− 0.61,-0.03)	− 0.24(− 0.53,0.06)	−0.30^*^(− 0.59,-0.01)
*p* value for trend	< 0.001	< 0.05	< 0.001	< 0.001	< 0.05	< 0.005

All models have adjusted for fixed effects factors (age and grade) and random effects factors (school and class).

* *p* < 0.05, † *p* < 0.01, ‡ *p* < 0.001

**Fig. 3:**
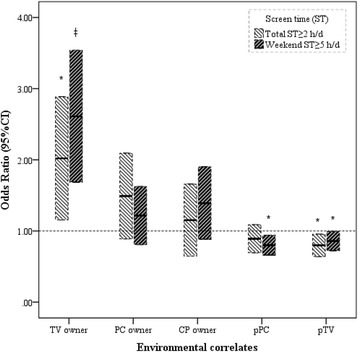
Odds ratios of environmental correlates on prolonged screen time in Chinese students. The independent variables in the logistic models included sex, age, grade, television, personal computer (PC), cell phone, presence of parents/others while playing (pPC) or watching TV (pTV). * *p* < 0.05, ‡ *p* < 0.001

Significant environmental correlates of prolonged ST (≥2 h/day) were having a TV in the bedroom (OR = 1.82, 95%CI: 1.15–2.87, *p* < 0.01) and presence of parents/others while watching TV (OR = 0.78, 95%CI: 0.64–0.95, *p* < 0.05) (Fig. 3). Meanwhile, TV (OR = 2.43, 95%CI: 1.68–3.53, *p* < 0.001), the presence of parents/others while playing computer (OR = 0.80, 95%CI: 0.67–0.95, *p* < 0.05), and the presence of parents/others while watching TV (OR = 0.85, 95%CI: 0.72–0.99, *p* < 0.05) were environmental correlates of prolonged ST on weekends (≥5 h/day).

## Discussion

Based on data collected using a validated questionnaire, male sex, junior high school students, weekends, presence of parents/others, and media accessibility were significantly associated with prolonged ST. These results are important for developing a series of effective strategies against prolonged ST in 8–19-year-old students in the future.

The percentage of prolonged weekday and weekend leisure ST (≥2 h/d) were 11.7% and 83.1%, respectively; this was lower for weekdays and greater for weekends compared with the results of another study in Chinese children [25]. The findings that boys have more leisure ST than girls do and that there is more ST on weekends than on weekdays are also consistent with previous studies [10, 25]. Another study indicated that age was positively associated with ST [10]. In our participants, age and grade were nonlinearly associated with ST (leisure ST decreased after 15 years of age/junior high school grade). This might be because there is more pressure in regards to studying and preparing for college entrance examinations in Chinese senior high school students than is seen for senior high school students in Western culture; this may push students to place more attention and time on study-related behavior, resulting in a “crowding-out effect” [26, 27].

In addition, correlations between environmental factors and leisure ST were not significantly changed after additional adjustment for parental education levels. In contrast with previous studies [28], low parental education was not a risk of prolonged ST in students in this study. In China, this might because parents with higher education level (high school or above) do not restrict their children’s SSB as well as because they might have greater media accessibility owing to a higher socioeconomic status [29].

Previous studies have shown that parents viewing TV with their children or parental role modeling of TV viewing were positively associated with the duration of TV viewing [30–32]. In our results, however, the presence of parents/others was negatively associated with leisure ST. Parents of students in the present study might play a supervisory role while watching TV with their children, rather than merely being present or accompanying their children. The results also potentially indicate that parental rules concerning screen viewing or parental expectations could mediate SSB (especially for TV viewing) in this population [26, 33]. Accordingly, media accessibility, especially having a TV set in the bedroom, seems a more important target than the accompaniment of parents/others for preventing prolonged leisure ST. These results differ from those of Carson et al. [34], who indicated that social environments might be more important than physical environmental factors, such as neighborhoods. Upon further exploration of the data, the presence of a TV set in the bedroom was not associated with leisure ST in our participants. Students with a TV in the bedroom did not have a significantly greater amount of time spent watching TV, but did have a greater amount of time using a phone/tablet and computer. Therefore, this does not appear to be a causal relationship, but rather just a relationship observed between the presence of a TV set in the bedroom and leisure ST. Most students with a TV in the bedroom were older and were more likely to be alone (not accompanied by parents or others) while watching TV. It was deduced that these students might have a greater amount of individual space, independence, and insensitivity to parental control, which could increase leisure ST.

Furthermore, our results indicate that cell phone/tablet-based leisure ST has become a main component of ST, although Jiang et al. reported that playing on a mobile phone was less prevalent before 2014 in Chinese adolescents [25]. In our data, 91.0% students with one type of screen and 96.7% students with two types of screens were the owners of a cell phone/tablet. This observation shows that cell phone/tablet-based leisure ST has dramatically increased in recent years and now comprises the largest proportion of ST, as was observed with Australian children [35]. These results also suggest a call for future studies that focus on exploring the correlates of small/mobile leisure ST because of its potentially different correlates.

This study has some limitations. First, caution should be noted in generalizing our results or inferring any causal relationships, because this was a cross-sectional study performed in the north of Zhejiang Province (a region with a relative high economic status in China), even though the students were from different grades and schools. Second, the presence of parents/others has not been defined clearly; for example, it is unclear whether there were efforts to restrict or encourage students’ SSB; moreover, the study lacked a direct investigation of parents. Previous studies indicated that parental ST, role modeling, and familial rules for SSB were related to leisure ST of students or children [36, 37]. The strength of our study was measuring time spent using cell phones/tablets, which was highly prevalent in Chinese students. Previous studies in this field mainly focused on descriptions of sociodemographic characteristics or correlates of TV viewing and computer use.

## Conclusions

Greater media accessibility and less presence of parents/others are associated with prolonged leisure ST in 8–19-year-old Chinese students, based on data collected using our reliable questionnaire. Additional attention should be paid to mobile/small screen-based devices, such as cell phones and pads/tablets, to avoid prolonged leisure ST, especially in boys and/or on weekends. Development of effective strategies for intervention of small/mobile device-based ST will be required in future studies.

## Abbreviations

CI: Confidence interval
OR: Odds ratio
SSB: Screen-based sedentary behavior
ST: Screen time
TV: Television
